# SARS-CoV-2 keeps evolving, so must our research efforts

**DOI:** 10.1093/nsr/nwaf138

**Published:** 2025-04-09

**Authors:** Wentai Ma, Kaiwen Zhang, Yunlong Cao, Mingkun Li

**Affiliations:** China National Center for Bioinformation, China; Beijing Institute of Genomics, Chinese Academy of Sciences, China; University of Chinese Academy of Sciences, China; China National Center for Bioinformation, China; Beijing Institute of Genomics, Chinese Academy of Sciences, China; University of Chinese Academy of Sciences, China; Biomedical Pioneering Innovation Center, Peking University, China; School of Life Sciences, Peking University, China; Changping Laboratory, China; China National Center for Bioinformation, China; Beijing Institute of Genomics, Chinese Academy of Sciences, China; University of Chinese Academy of Sciences, China

Although public attention to SARS-CoV-2 has waned, the virus continues to circulate within the population and continues to mutate and cause periodic surges. Mutations could alter the virus's antigenic properties, facilitate immune escape [1] and lead to persistent prevalence and recurrent outbreaks. Additionally, mutations could potentially enhance the virus's transmissibility and pathogenicity [2,3], thereby posing an ongoing threat to public health.

Studies on SARS-CoV-2 have surpassed those on all other viruses in terms of the number of published articles. However, our understanding of the virus's evolution remains insufficient. Identifying high-risk variants from surveillance data and predicting how the virus evolves to produce new variants remains challenging.

**Figure 1.**
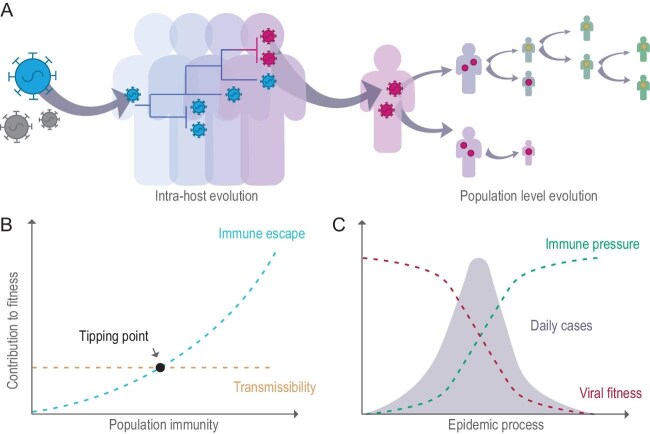
Evolution trajectory of SARS-CoV-2. (A) Evolution of SARS-CoV-2 at the intra-host and population level. (B) Dynamics of forces driving the evolution of SARS-CoV-2. (C) Dynamics of viral fitness and immune pressure on the virus (without mutations) over time.

Mutation in the virus initially emerges as single or limited copies during its replication within an infected host, due to errors introduced by the viral RNA-dependent RNA polymerase (RdRp) or through RNA editing mechanisms (Fig. 1A). The frequency of intra-host mutations can fluctuate significantly over time, with some variants becoming fixed within the host. However, the factors driving their fixation remain poorly understood. In immunocompromised patients with prolonged infections, the virus evolves toward resistance to serum neutralization, indicating that these mutations may be driven by immune pressure. Notably, when multiple viral haplotypes coexist within an individual—whether through *de novo* mutations or coinfection of different strains—recombination can occur, further increasing the diversity of intra-host variants [4]. However, not all viral haplotypes are transmitted to subsequent hosts, as transmission involves an estimated bottleneck of just 1 to 10 viral particles [5]. While a higher intra-host frequency increases the likelihood of shedding from the donor, successful infection also depends on the virus's ability to evade immunity, enter, and replicate in host cells—pressures that may differ from those acting on the virus within the host [6].

The evolutionary history of SARS-CoV-2 indicates that selection at the population level primarily targets immune evasion and transmissibility, with the latter being influenced by the virus's ability to bind to the ACE2 receptor, replication efficiency, spike protein conformation, proteolytic cleavage, and the viral fusion process [7]. Immune pressure evolves over time due to the accumulation of population immunity from vaccination and prior infections, leading to shifts in selective preferences (Fig. 1B). Particularly, when a novel virus with antigenic differences from previous viruses emerges in the population, its evolution is initially largely unaffected by immune pressure due to the absence of pre-existing humoral immunity. However, over time, as the variant's prevalence increases, the selection pressure gradually shifts towards immune evasion. This leads to a situation where a variant's fitness advantage becomes closely tied to its prevalence [8]. Despite this understanding, the precise quantitative relationships, including the tipping point where immune pressure becomes the primary driver of viral evolution, remain unclear. (Fig. 1B and C).

Due to distinct immune backgrounds across populations, shaped by variations in infection and/or vaccination histories, the virus evolves along varying trajectories in different populations or regions [9]. Variants that show growth advantages in one country may not necessarily be favored in others. Additionally, chronic infections, which account for 0.1%–0.5% of all infections and typically occur in immunocompromised individuals, can prolong the presence of the virus for more than two months [10]. This extended infection period accelerates viral evolution, potentially resulting in significant evolutionary leaps. Furthermore, re-spillover from animals to humans may introduce new mutations that facilitate adaptation to new animal hosts.

Predicting the next pandemic variants could be regarded as the ‘Crown Jewel’ of viral evolution research. Successful prediction would enable us to prepare for the next pandemic in advance, reducing virus spread, preventing healthcare system overload, and accelerating the development of long-acting, broad-spectrum vaccines and drugs that are less likely to be evaded by future variants. Notably, this same question has been investigated for seasonal influenza for over two decades, yet it remains only partially resolved [11]. Despite more than 2000 scientific papers on ‘SARS-CoV-2’ and ‘prediction’ in the PubMed database—twice the number of papers on ‘influenza’ and ‘prediction’—we are still far from accurately forecasting the next SARS-CoV-2 variants.

Predictions of future dominant variants typically prioritize genomic sites with critical viral function, such as immune evasion or transmissibility. This task can be divided into two categories: pre-variant prediction and post-variant prediction. Pre-variant prediction refers to forecasting future dominant variants that have not yet emerged, while post-variant prediction involves evaluating the fitness of newly emerged variants. The former is more challenging, as it requires predicting which mutations will occur among numerous potential options that could similarly enhance viral fitness. While many evolutionary driving forces and constraints have been identified, such as immune evasion, ACE2 binding affinity, deamination, protein stability and codon preference [7], the list is still incomplete, and the quantitative relationships between these factors and mutation occurrence remain unclear. This uncertainty complicates efforts to estimate the likelihood of mutations.

In predicting which variants are likely to become prevalent in the population, existing studies predominantly prioritize immune evasion—specifically, the hypothesis that a variant's fitness is determined by its relative capacity to evade host immunity compared to co-circulating variants [12]. However, epidemiological data reveal that prevalent variants are not invariably those with the highest potential to evade immune pressure [9], suggesting that additional factors may critically influence variant success. Moreover, the immune landscape within host populations is dynamic, necessitating continuous monitoring of immune background, which is both resource-intensive and time-consuming.

Current assessments of viral fitness primarily focus on the receptor-binding domain (RBD) of the Spike protein, which is essential for viral entry into host cells and a key target for host immunity. However, other genomic regions, such as the N and Orf9b proteins, also play roles in suppressing the innate immune response [3,13]. Notably, high-throughput functional screening methods for these regions are lacking. Additionally, SARS-CoV-2 evolution does not always follow a linear pattern as chronic infections, recombination and cross-species spillover can contribute to unpredictable evolutionary leaps. The presence of epistatic effects, where interactions between different mutations influence their combined impact, further complicates the assessment of mutations’ effects on fitness [2].

The extensive accumulation of SARS-CoV-2 genome sequences, epidemiological data and experimental findings over the past five years has offered an unparalleled insight into the evolutionary trajectory of this pathogen. These data have catalyzed the development of new methods, resources and evolutionary frameworks applicable to both individual and population-level analyses, some of which extend beyond SARS-CoV-2 to other viruses. Sustained genomic surveillance remains imperative to monitor emerging variants, complemented by population-level immune monitoring through longitudinal serological studies. This dual surveillance strategy is critical for detecting antigenic escape variants capable of evading humoral immunity and circumventing therapeutic interventions, which may lead to new pandemic waves and pose risks to vulnerable populations [8]. Furthermore, as viral evolutionary pathways remain dynamic and context-dependent, expanding data sets will progressively refine predictive models of adaptation.

The prediction of SARS-CoV-2 evolution has been greatly accelerated by functional data from rapidly advancing high-throughput screening technologies. Nevertheless, systematic characterization of mutations effects—especially those located outside the S gene and those interacting with other mutations—still await breakthroughs. Recent advances in AI offer transformative potential by leveraging evolutionary and structural data to predict the functional effects of individual mutations and their combinations [14,15]. While the growing body of multivariate data from SARS-CoV-2 research provides valuable insights, the lack of clarity regarding how these data interconnect hampers accurate estimations of a mutation's contribution to viral fitness. AI's capacity to unify heterogeneous data sets could simplify this integration, uncovering interaction networks between viral properties, and enabling more precise fitness estimates. Additionally, the generative capabilities of AI models enable systematic exploration of viral mutation landscapes, including simulating both observed and unobserved mutation combinations, enabling better prediction of potential future mutations.

The evolution of viruses continuously generates new variants with differing functional potentials, yet intrinsic fitness alone does not guarantee widespread prevalence. Environmental factors, such as mismatched population immunity, can cause immune-escape variants to be outcompeted by co-circulating strains with higher transmissibility, leading to their disappearance or causing them to persist at low levels until favorable conditions arise. The complex relationship between spatiotemporal environment factors, viral evolution and viral prevalence remains largely unknown, posing significant challenges in predicting whether and when a new variant could trigger a new wave of epidemics, as well as the duration of its circulation and the strength of its peak. Recently, new models that integrate viral mutations into classical Susceptible-Infected-Removed (SIR) epidemiologic frameworks have emerged, demonstrating promising results [16]. Future research will introduce additional parameters and refine these models to improve their accuracy.

There is an ongoing debate about whether the virus will become more virulent or milder in the future. Increased virulence does not necessarily make a virus less adaptive, as evidenced by the Delta variant, which is more pathogenic than earlier variants. Nevertheless, assessing the virulence of new variants has become a high-priority task for further studies, which currently rely heavily on clinical reports and time-consuming lab experiments. High-throughput screening assays, rapid experimental tests and efficient computational tools should be the new focus of future research [17].

With advancements in technology and the ongoing efforts of scientists, we will undoubtedly gain new insights into SARS-CoV-2—an ever-evolving virus—that will help us better prevent and treat this disease.
